# Correlation Between Maximal Tongue Pressure and Swallowing Function in Spinal and Bulbar Muscular Atrophy

**DOI:** 10.3389/fneur.2021.704788

**Published:** 2021-09-01

**Authors:** Dae-Won Gwak, Seung-Hwan Jung, Yu-Sun Min, Jin-Sung Park, Hee-Jin Cho, Donghwi Park, Min Woo Hong, Min-Gu Kang

**Affiliations:** ^1^Department of Rehabilitation Medicine, School of Medicine, Kyungpook National University, Kyungpook National University Chilgok Hospital, Daegu, South Korea; ^2^Department of Neurology, School of Medicine, Kyungpook National University, Kyungpook National University Chilgok Hospital, Daegu, South Korea; ^3^Department of Physical Medicine and Rehabilitation, Ulsan University Hospital, University of Ulsan College of Medicine, Ulsan, South Korea; ^4^Department of Physical Medicine and Rehabilitation, Dong-A University College of Medicine, Dong-A University Hospital, Busan, South Korea

**Keywords:** bulbo-spinal atrophy X-linked, motor neuron disease, neurodegenerative diseases, tongue, deglutition disorders

## Abstract

**Background:** Spinal and bulbar muscular atrophy (SBMA) is an X-lined motor neuron disease characterized by progressive muscle weakness, bulbar palsy, and dysphagia. Dysphagia is associated with tongue weakness, which is a common manifestation of SBMA. This study aimed to investigate the correlations between tongue pressure and dysphagia in patients with SBMA.

**Materials and Methods:** Thirty-nine genetically confirmed SBMA patients underwent a videofluoroscopic swallowing study (VFSS) and tongue pressure assessment. Then, we analyzed the maximal tongue pressure (MTP), oral transit time, penetration-aspiration scale (PAS), videofluoroscopic dysphagia scale (VDS), amyotrophic lateral sclerosis functional rating scale-revised (ALSFRS-R), and 6-min walk test (6MWT). Pearson and Spearman correlation coefficients were calculated to analyze the association of the MTP with clinical, swallowing, and functional parameters.

**Results:** In the correlation analysis, MTP was negatively correlated with disease duration (*r* = −0.396, *p* = 0.013) and VDS (*r* = −0.426, *p* = 0.007), and positively correlated with ALSFRS-R (*r* = 0.483, *p* = 0.002) and 6MWT (*r* = 0.396, *p* = 0.013). The bulbar (*r* = 0.367, *p* = 0.022) and gross motor (*r* = 0.486, *p* = 0.002) domains of the ALSFRS-R were correlated with MTP.

**Conclusion:** Tongue pressure assessment can be used as a safe and easy tool to assess swallowing function in SBMA patients. Moreover, MTP reflects functional states, including activities of daily living and gait performance, showing it to be a potential biomarker for physical performance in SBMA.

## Introduction

Spinal and bulbar muscular atrophy (SBMA) is an X-linked neurodegenerative disease caused by an expansion of the CAG repeat sequence in the first exon of the androgen receptor (AR) gene (1–3). It is characterized by progressive proximal limb and bulbar weakness, dysphagia, and generalized fasciculations, predominantly of the facial muscles (4, 5). Among these symptoms, dysphagia contributes to aspiration pneumonia, which is the primary cause of death in SBMA (6).

The tongue plays an essential role in swallowing. During the oral phase, the tongue forms and holds a bolus on the anterior tongue. At this time, the posterior tongue prevents the food from going into the pharynx prematurely. After bolus formation, the posterior transport of the bolus is initiated by the anterior tongue pressing against the hard palate (7). In the pharyngeal phase, the posterior tongue retracts against the pharyngeal wall and contributes to the downward propulsion of the bolus. Because of these vital functions of the tongue, sufficient strength of the tongue is essential for healthy swallowing (8).

Reduced tongue pressure is associated with the development of dysphagia symptoms (9). Previous studies have suggested an association with various diseases, including stroke, sarcopenia, and Parkinson's disease (10–12). In patients with SBMA, the atrophy and weakness of the tongue are common early findings (13, 14). Involvement of the motor nuclei located in the brainstem causes the characteristic bulbar muscle weakness and atrophy (15). The bulbar muscle weakness leads to the typical patterns of dysphagia in about 80% of patients with SBMA, including decreased bolus clearance and vallecular residue after swallowing (16, 17). Therefore, the evaluation of tongue strength can lead to an estimate of the swallowing function in SBMA patients. Moreover, since tongue pressure decreases early in the disease, tongue pressure measurement will be helpful for early detection in SBMA.

This study aimed to investigate the correlation between tongue pressure and the severity of dysphagia in patients with SBMA. Furthermore, we investigated the association between tongue pressure and physical function to determine the possibility of tongue pressure as a biomarker for physical performance.

## Materials and Methods

### Study Design

This was a retrospective review study of SBMA patients admitted to the Kyungpook National University Chilgok Hospital between January 2019 and June 2020. This study was approved by the Institutional Review Board of Kyungpook National University Chilgok Hospital (No. 2020-10-007). Ethical approval was waived in view of the retrospective nature of the study.

### Participants

Patients with a genetically confirmed SBMA diagnosis were retrospectively identified from our hospital's videofluoroscopic swallowing study (VFSS) database from January 2019 to June 2020. The inclusion criteria were: (1) age ≥ 20 years, (2) at least 38 CAG repeats in the androgen receptor gene, (3) tongue pressure assessment performed on the same day of VFSS, and (4) clinical diagnosis of SBMA with more than one of the following symptoms: muscle weakness, muscle atrophy, bulbar palsy, hand tremor, and dysphagia. The exclusion criteria were: (1) any history of androgen suppression therapy, and (2) presence of other dysphagia-related diseases, such as stroke or nasopharyngeal cancer.

### Swallowing Function

Swallowing evaluation was conducted using videofluoroscopy with a modified Logemann protocol. Participants were seated upright on a chair, and nasogastric tubes were removed before the study. Test diets included diluted barium (35% w/v), curd-type yogurt, a semi-blended diet, and boiled rice. Images were recorded at a speed of 30 frames per second using a digital capture card (BR278; Beautifulrecorder, Guangzhou, China). Recorded images were analyzed frame-by-frame and scored based on the videofluoroscopic dysphagia scale (VDS) and penetration-aspiration scale (PAS). Two experienced physiatrists with more than 2 years' experience in VFSS interpretation verified the results.

The VDS is a validated tool to predict the prognosis of dysphagia in various etiologies. It consists of 14 items: lip closure, bolus formation, mastication, apraxia, tongue-to-palate contact, premature bolus loss, oral transit time, triggering of pharyngeal swallow, vallecular residue, laryngeal elevation, pyriform sinus residue, coating of pharyngeal wall, pharyngeal transit time, and aspiration. The total score ranges from 0 to 100, with higher scores indicating worse swallowing function (18). Lip closure, bolus formation, mastication, and tongue-to-palate contact were scored on three levels as follows: normal, inappropriate, or none. Apraxia was scored as follows: none, mild, moderate, or severe. The amount of premature bolus loss was classified into four levels: none, <10%, 10–50%, or >50% of bolus. Oral transit time was scored on two levels: ≤ 1.5 or >1.5 s. Triggering of pharyngeal swallow was graded on two levels: normal or delayed. The amount of vallecular residue, and pyriform sinus residue were classified into four levels: none, <10%, 10–50%, or >50% of the area of the vallecular or pyriform sinus in two-dimensional view. Laryngeal elevation was classified into two levels: normal or impaired. Coating of pharyngeal wall was graded on two levels: absent or present. Pharyngeal transit time was scored on two levels: ≤ 1.0 or >1.0 s. Aspiration was graded on three levels: none, supraglottic penetration, or subglottic aspiration.

The PAS is an 8-point scale for identifying the severity of penetration or aspiration, with higher scores representing a greater risk of aspiration (19). This is determined by the depth of material invasion into the airway and whether the effort is provided to expel the material. Epiglottic retroflexion was evaluated based on the findings of VFSS. Decreased epiglottic retroflexion was defined as a diminished degree of vertical to horizontal epiglottic movement during swallowing.

We divided participants into subgroups based on VDS ( ≤ 24: less severe dysphagia; > 24: more severe dysphagia), PAS (1~2: non-penetrator/aspirators; 3~8: penetrator/aspirators), and epiglottic retroflexion (normal vs. decreased epiglottic retroflexion). The median value of VDS was 24, which was chosen as the cut-off point.

### Tongue Pressure

Maximal tongue pressure (MTP) was measured using TPS-100 (CyberMedic, Iksan, South Korea) on the same day as the VFSS. It was measured *via* oral pressures generated against the air bulb of the device. Patients were asked to compress the air bulb on their tongue against the anterior region of hard palate for 3 s as hard as possible, and the maximal value was recorded. After three trials with intervals of 1 min, the average of the maximal pressures was selected for the analysis. Participants underwent two sets of assessments before and after the VFSS, respectively, and the result of the first set was used in the analysis as the MTP.

### Functional Evaluation

Functional measures included the amyotrophic lateral sclerosis functional rating scale-revised (ALSFRS-R), and a 6-min walk test (6MWT). The ALSFRS-R is a validated questionnaire-based, 12-item functional scale (20). It is divided into four domains: bulbar, gross motor, fine motor, and respiratory. The total score of ALSFRS-R ranges from 0 to 48, and higher scores indicate better function. The 6MWT is a reliable measure of functional capacity in SBMA. It was conducted according to a previous study to evaluate gait function and endurance (21).

### Statistical Analysis

Pearson's and Spearman correlation analyses were used to analyze the correlations of MTP with clinical, swallowing, and functional parameters. Pearson's correlation coefficient was calculated for normally distributed continuous variables, and spearman's correlation coefficient was calculated for non-normal distributions. One-way ANOVA with Tukey HSD *post-hoc* test was used to compare the swallowing parameters among the different disease duration groups. Continuous variables were compared between two groups with the use of Student's *t*-test. Within-day test–retest reliability was analyzed by computing the intraclass correlation (ICC). Statistical significance was accepted when a *p*-value was < 0.05. Statistical analyses were performed using the statistical software R version 3.6.0 (R Foundation for Statistical Computing, Vienna, Austria).

## Results

Table 1 shows the clinical characteristics of the participants. A total of 39 genetically confirmed SBMA patients were analyzed. The mean value of MTP was 24.0 kPa. Within-day test–retest reliability showed excellent test–retest reliability (ICC = 0.93, 95% CI = 0.89–0.96, *p* < 0.001).

**Table 1:** Clinical characteristics of the participants (*n* = 39).

**Characteristics**	**Value**
Male	39 (100%)
Age (years)	54.0 ± 9.3
Disease duration (years)	10.8 ± 5.1
CAG repeat number	45.8 ± 3.9
Maximal tongue pressure (kPa)	24.0 ± 8.4
Penetration-aspiration scale	2.6 ± 1.5
Videofluoroscopic dysphagia scale	
Oral phase	6.1 ± 4.9
Pharyngeal phase	21.1 ± 10.6
Total score	27.4 ± 12.6
ALSFRS-R	39.6 ± 3.7
6-min walk test (m)	272.9 ± 163.1

*Variables are presented as number (%) or mean ± standard deviation*.

*ALSFRS-R, Amyotrophic Lateral Sclerosis Functional Rating Scale-Revised*.

### Relationships Between Disease Duration and Swallowing Function

**Figure 1:**
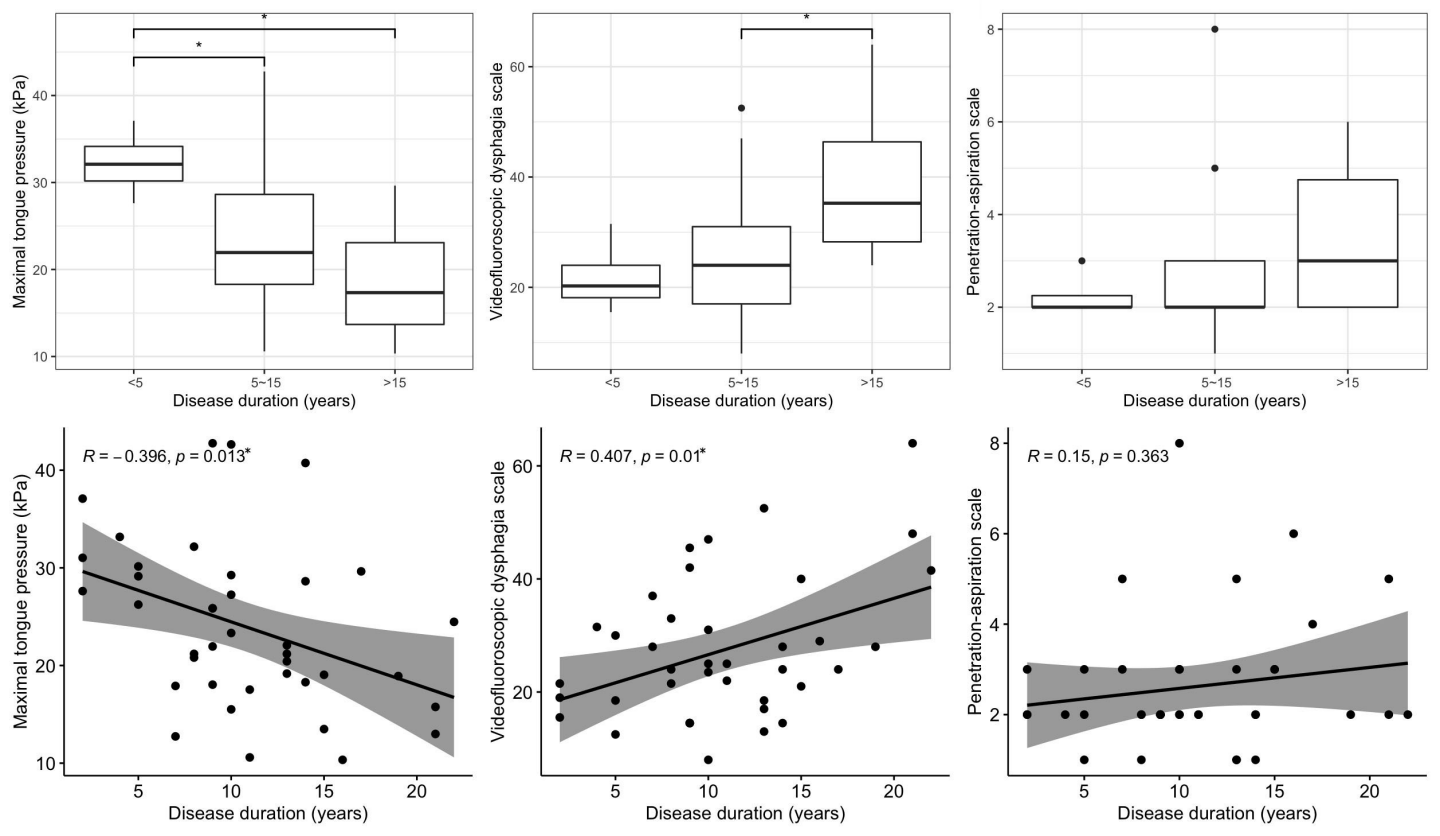
Relationships between disease duration and swallowing parameters. *p < 0.05.

Figure 1 shows the relationships between disease duration and swallowing parameters, including MTP, VDS, and PAS. Disease duration was associated with MTP (*r* = −0.396, *p* = 0.013) and VDS (*r* = 0.407, *p* = 0.010), but not with PAS (*r* = 0.150, *p* = 0.363). As the duration of disease increased, MTP and VDS worsened. Unlike VDS, MTP was significantly different between patients with a disease duration of <5 years and those with a disease duration of 5–15 years (*p* = 0.019).

### Relationships Between Maximal Tongue Pressure and Clinical Characteristics, Swallowing, and Functional Measures

Table 2 shows the correlation coefficients of MTP with clinical characteristics, swallowing, and functional parameters. MTP was correlated with disease duration (*r* = −0.396, *p* = 0.013), but not with age and CAG repeat number. There were significant negative correlations between MTP and the VDS oral phase (*r* = −0.382, *p* = 0.016), VDS pharyngeal phase (*r* = −0.324, *p* = 0.044), VDS total score (*r* = −0.426, *p* = 0.007), and PAS (*r* = −0.321, *p* = 0.047). Besides swallowing parameters, MTP was also well-correlated with other functional measures. MTP was positively correlated with ALSFRS-R (*r* = 0.483, *p* = 0.002) and the 6MWT (*r* = 0.396, *p* = 0.013). Bulbar and gross motor domains were significantly correlated with MTP in the analysis of subdivisions of ALSFRS-R (*r* = 0.367, *p* = 0.022, *r* = 0.486, and *p* = 0.002); meanwhile, fine motor and respiratory domain was not (*r* = 0.289, *p* = 0.075, *r* = 0.279, and *p* = 0.085).

**Table 2:** Correlations of maximal tongue pressure with clinical parameters and functional measures.

**Parameter**	**Correlation coefficient**	***P*-value**
Age	−0.302	0.061
Disease duration	−0.396	0.013^*^
CAG repeat number	0.143	0.386
Videofluoroscopic dysphagia scale		
Oral phase	−0.382	0.016^*^
Pharyngeal phase	−0.324	0.044^*^
Total score	−0.426	0.007^*^
Penetration-aspiration scale	−0.321	0.047^*^
6-min walk test (m)	0.396	0.013^*^
ALSFRS-R		
Bulbar	0.367	0.022^*^
Fine motor	0.289	0.075
Gross motor	0.486	0.002^*^
Respiratory	0.279	0.085
Total score	0.483	0.002^*^

*ALSFRS-R, Amyotrophic Lateral Sclerosis Functional Rating Scale-Revised*.

* *p < 0.05*.

**Figure 2:**
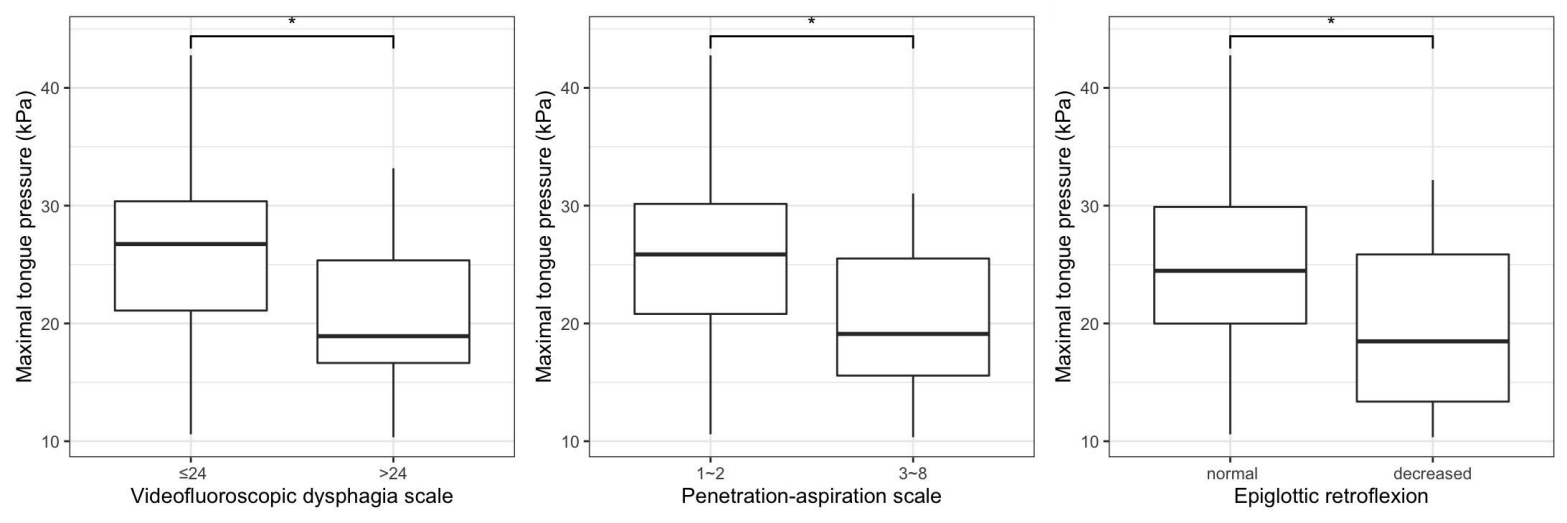
Relationships between maximal tongue pressure and swallowing function. *p < 0.05.

**Figure 3:**
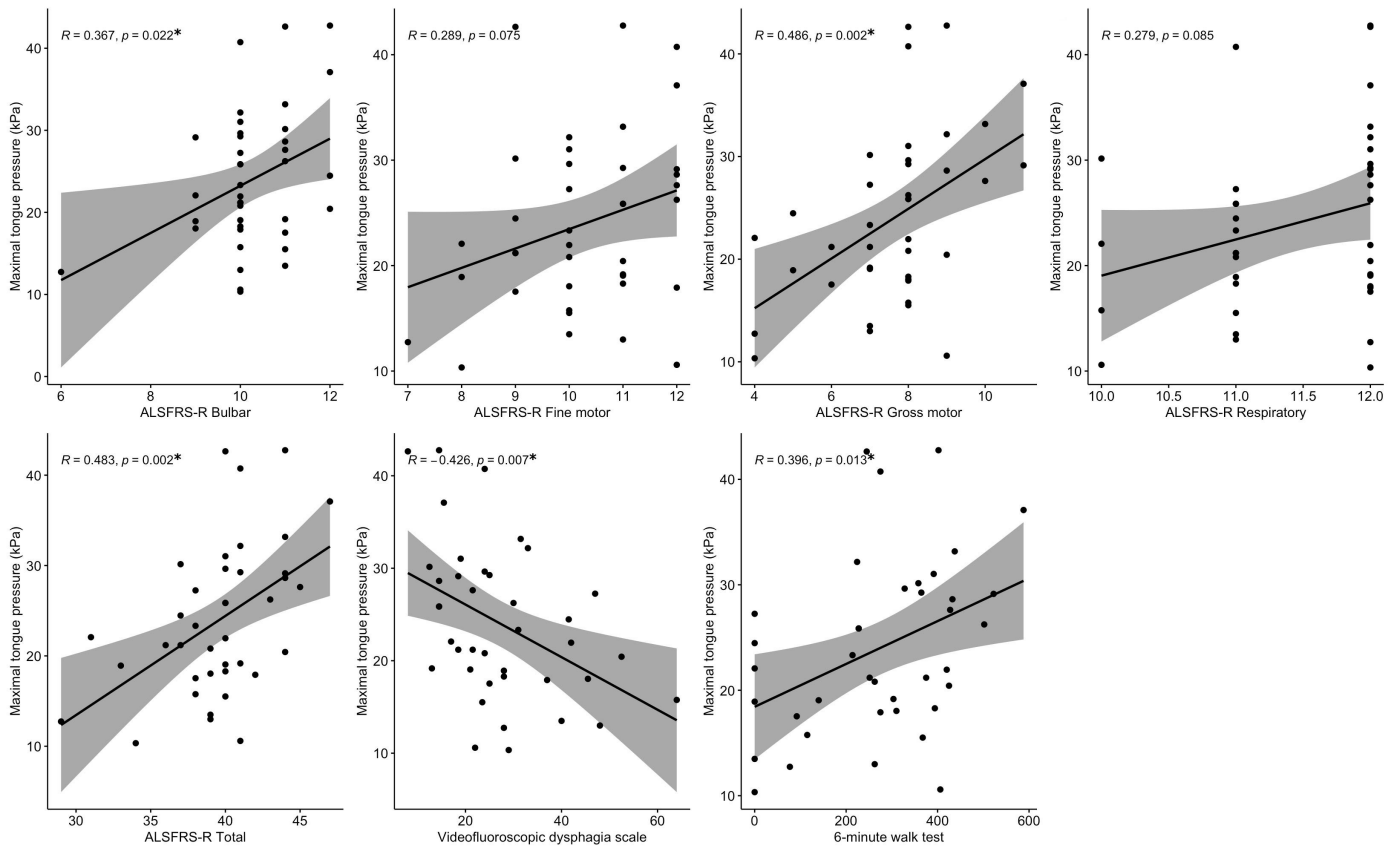
Scatter plots of maximal tongue pressure and functional parameters. *p < 0.05.

Figure 2 shows the relationships between MTP and VDS, PAS, and epiglottic retroflexion. MTP was significantly lower in patients with a VDS higher than 24 compared to patients with a VDS of 24 or less (*p* = 0.020). Significantly greater MTP was shown in non-penetrator/aspirators than in penetrator/aspirators (*p* = 0.039). The normal epiglottic retroflexion group showed statistically greater MTP than the decreased epiglottic retroflexion group (*p* = 0.023). Figure 3 shows the scatter plots of maximal tongue pressure and functional measures.

## Discussion

This study revealed that MTP was negatively associated with disease duration, VDS, and PAS in patients with SBMA, which suggests that it decreases with disease progression and reflects swallowing function. Moreover, MTP was positively associated with the bulbar and gross motor domains of the ALSFRS-R and the 6MWT, which implies that MTP also reflects physical performance in patients with SBMA.

Mano et al. (22, 23) suggested that tongue pressure is a reliable biomarker of swallowing function in SBMA patients. That study reported significant relationships between tongue pressure and swallowing parameters mainly based on patient responses to questionnaires, such as the swallowing quality of life (SWAL-QOL) and swallowing disturbance questionnaire-Japanese (SDQ-J). The novelty of our study is that it showed significant correlations between MTP and objective swallowing measures, including VDS, PAS, and epiglottic retroflexion based on the findings of VFSS. This is consistent with the previous findings that SBMA patients' MTP reflects swallowing function, and specifically confirmed that the tongue pressure is reduced early on.

As the epiglottis folds back, the arytenoids tilt forward and come into contact with the base of the epiglottis, blocking the entrance to the larynx (24). At this time, the food bolus slides down the epiglottis and descends into the pyriform sinus. This is a critical mechanism to protect the airway (25). Tongue muscles play a key role in epiglottic retroflexion. Contraction of the styloglossus and hyoglossus muscles pulls the tongue posteriorly to contact the pharyngeal wall (26, 27). This enables the passive retroflexion of the epiglottis to invert horizontally (28). Therefore, adequate tongue strength is essential in airway protection. In line with this explanation, patients with normal epiglottic retroflexion showed significantly greater tongue pressure than those with decreased epiglottic retroflexion in the present study.

The correlation of MTP with disease duration showed multiple outliers. This may be because the onset of bulbar weakness or dysphagia in SBMA varies (6). According to the previous study, the association between MTP and disease duration was not necessarily related in patients without subjective dysphagia, which may also explain the outliers (22). Although the correlations of MTP with the bulbar subscore of the ALSFRS-R and the 6MWT were significant, they were weak. The bulbar subscore of the ALSFRS-R consists of three items: speech, drooling, and dysphagia. These functions are related to tongue strength; however, much more diverse factors are involved. Also, as can be seen from the scatter plot, the bulbar subscores of the participants were all nine or more, indicating that most of the participants had mild deterioration of bulbar function. This may have led to the weak correlation. The deterioration degree of bulbar function and overall muscle strength may not match in some patients, and this variability of phenotype may have contributed to the weak correlation between the MTP and the 6MWT.

Swallowing evaluations such as VFSS or fiberoptic endoscopic evaluation of swallowing (FEES) require expensive facilities and highly trained specialists. Hospitals equipped with such facilities are not common; therefore, such facilities are often located at long distances from patients' residences, which makes it difficult for SBMA patients to visit hospitals regularly. VFSS poses a risk of radiation exposure, FEES can cause discomfort when the endoscope is inserted into the nostril, and neither can avoid the risk of aspiration (29, 30). On the other hand, tongue pressure assessment is a safe and easy tool without radiation exposure or discomfort. Moreover, unlike VFSS and FEES, which are qualitative evaluations, tongue pressure assessment is a quantitative method, not requiring experts to interpret.

This study had several limitations. First, this study did not enroll a control group. In a recent study, MTP was measured using TPS-100 in community-dwelling older adults aged 65 years or more (31). Although the study participants were older than our study participants, they had an average MTP of 36.55 ± 3.29 kPa, which was higher than our study's average MTP of 24.0 ± 8.4 kPa. This was higher than the mean MTP of 26.99 ± 9.27 kPa in patients without subjective dysphagia in our study participants. This indirect comparison suggests that the MTP of SBMA patients may be lower than in normal subjects. Second, the study population consisted of patients within only one university hospital, which limits the generalizability of the results. Third, the sample size was relatively small because SBMA is a rare disease (32). Despite the limitations, the present study indicates the clinical usefulness of tongue pressure evaluation in patients with SBMA. Further long-term, multicenter study is recommended.

## Conclusion

MTP was significantly associated with objective confirmed findings of dysphagia severity. Moreover, it reflected activities of daily living and gait function in patients with SBMA. Therefore, tongue pressure assessment can be used as a safe and easy tool to assess the swallowing function of SBMA patients, with the potential to monitor disease progression.

## Data Availability Statement

The raw data supporting the conclusions of this article will be made available by the authors, without undue reservation.

## Ethics Statement

The studies involving human participants were reviewed and approved by the Institutional Review Board of Kyungpook National University Chilgok Hospital. Written informed consent for participation was not required for this study in accordance with the national legislation and the institutional requirements.

## Author Contributions

Y-SM, J-SP, DP, and M-GK: study concept and design. D-WG, S-HJ, and H-JC: collection and analysis of data. D-WG, S-HJ, Y-SM, J-SP, H-JC, DP, MWH, and M-GK: interpretation of study data. D-WG, S-HJ, and M-GK: drafting the manuscript. D-WG, S-HJ, MWH, and M-GK: editing the manuscript. All authors contributed to the article and approved the submitted version.

## Conflict of Interest

The authors declare that the research was conducted in the absence of any commercial or financial relationships that could be construed as a potential conflict of interest.

## Publisher's Note

All claims expressed in this article are solely those of the authors and do not necessarily represent those of their affiliated organizations, or those of the publisher, the editors and the reviewers. Any product that may be evaluated in this article, or claim that may be made by its manufacturer, is not guaranteed or endorsed by the publisher.
